# Supplementary material for: PROTAC-Mediated Dual Degradation of BCL-xL and BCL-2 Is a Highly Effective Therapeutic Strategy in Small-Cell Lung Cancer
Source: Cells. 2024 Mar 17;13(6):528. doi: 10.3390/cells13060528 (PMC10968744; doi:10.3390/cells13060528)
Supplement: Supplementary file 1 [file cells-13-00528-s001.zip › cells-2918800-supplementary.pdf]

SUPPLEMENTARY INFORMATION

PROTAC-mediated dual degradation of BCL-xL and BCL-2 is a highly effective therapeutic strategy in small-cell lung cancer

Khan *et al*.

SUPPLEMENTARY FIGURES

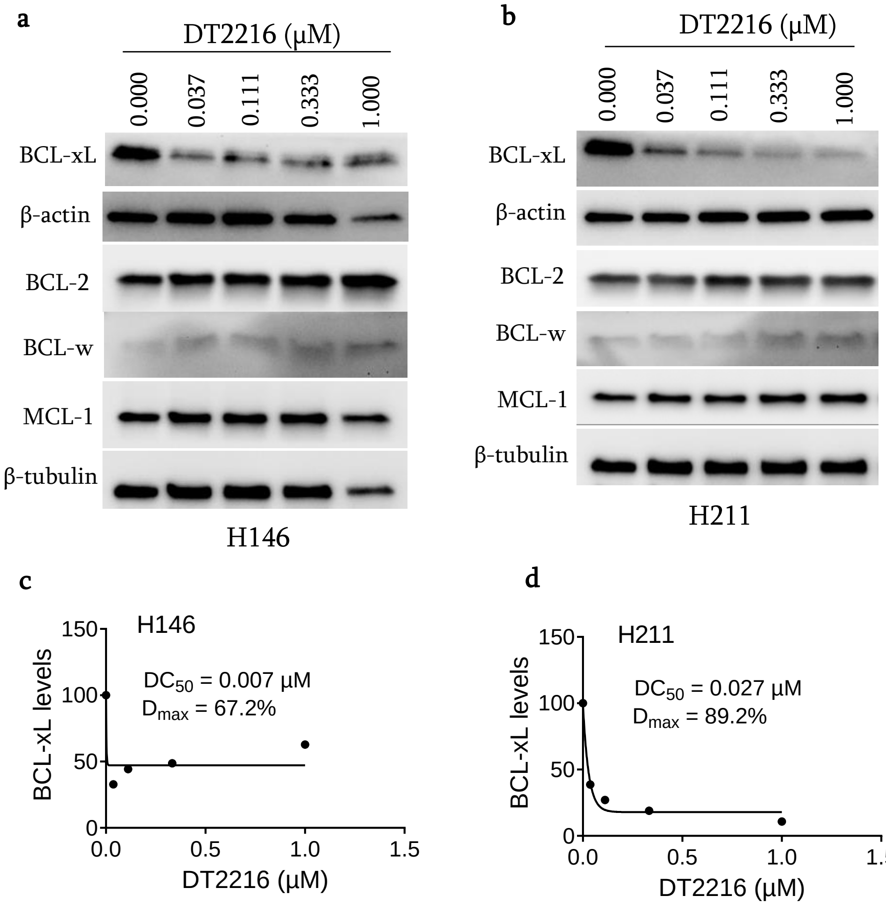

Supplementary Figure S1. DT2216 degrades BCL-xL in SCLC cells. **(a,b)** Immunoblot analyses of BCL-xL, BCL-2, BCL-w and MCL-1 in H146 (**a**) and H211 (**b**) cells after they were treated with increasing concentrations of DT2216 for 48 h. β-actin and β-tubulin were used as equal loading controls. **(c,d)** Densitometric analysis of BCL-xL along with DC_{50} and D_{max} values in H146 (**c**) and H211 (**d**) cells are shown.

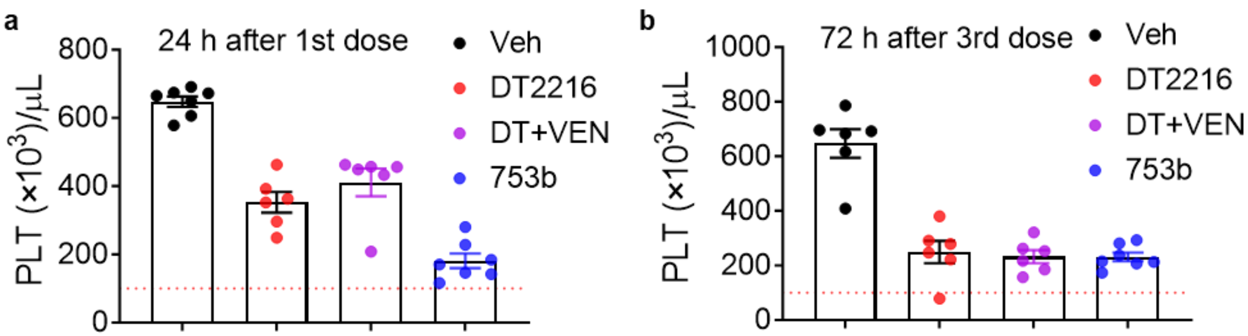

Supplementary Figure S2. 753b did not cause appreciable platelet toxicity in mice**. (a**) Enumeration of platelets (PLT) in mice blood 24 h after the 1st dose with vehicle, DT2216, DT2216+venetoclax, or 753b. Data are presented as mean ± SEM (*n* = 7, 6, 6, and 7 mice in vehicle, DT2216, DT2216 + venetoclax, and 753b groups, respectively). (**b**) Enumeration of platelets in mice blood 72 h after the 3rd weekly dose with vehicle, DT2216, DT2216+venetoclax, or 753b. Data are presented as mean ± SEM (*n* = 7, 6, 6, and 7 mice in vehicle, DT2216, DT2216 + venetoclax, and 753b groups, respectively).

Supplementary Table S1. Antibodies used in immunoblotting.

**Antibody**	**Clone**	**Antibody isotype**	**Catalog #**	**Concentration**
BCL-xL	_	Rabbit IgG polyclonal	2762	1:1000
BCL-2	50E3	Rabbit IgG monoclonal	2870	1:500
MCL-1	D35A5	Rabbit IgG monoclonal	5453	1:1000
BCL-w	31H4	Rabbit IgG monoclonal	2724	1:500
PARP/Cleaved PARP	46D11	Rabbit IgG monoclonal	9532	1:1000
Full-length caspase-3	_	Rabbit IgG polyclonal	9662	1:1000
cleaved caspase-3	_	Rabbit IgG polyclonal	9661	1:1000
β-tubulin	_	Rabbit IgG polyclonal	2146	1:3000
β-actin	D6A8	Rabbit IgG monoclonal	8457	1:5000
Secondary antibody		Anti-rabbit IgG, HRP	7074	1:3000

Footnotes: All the antibodies were purchased from Cell Signaling Technology, Danvers, MA.
